# Mobile Sensor Networks for Inspection Tasks in Harsh Industrial Environments

**DOI:** 10.3390/s100301599

**Published:** 2010-03-01

**Authors:** Jacob Mulder, Xinyu Wang, Franke Ferwerda, Ming Cao

**Affiliations:** 1 Logica, Eemsgolaan 1, P.O. box 70237, 9704 AE Groningen, the Netherlands; 2 Institute for Technology, Engineering & Management, Faculty of Mathematics and Natural Sciences, University of Groningen, Nijenborgh 4, 9747 AG Groningen, the Netherlands; E-Mails: x.wang.11@student.rug.nl (X.W.); f.ferwerda@student.rug.nl (F.F.); m.cao@rug.nl (M.C.)

**Keywords:** mobile sensor network, autonomous robot, industrial sensing, coordination algorithm

## Abstract

Recent advances in sensor technology have enabled the fast development of mobile sensor networks operating in various unknown and sometimes hazardous environments. In this paper, we introduce one integrative approach to design, analyze and test distributed control algorithms to coordinate a network of autonomous mobile sensors by utilizing both simulation tools and a robotic testbed. The research has been carried out in the context of the mobile sensing project, PicoSmart, in the northern provinces of the Netherlands for the inspection of natural gas pipelines.

## 1. Introduction

In recent years, there have been fast developments in mobile sensor networks for various applications, such as environment monitoring, infrastructure security and traffic control [1,2]. In particular, an emerging trend is to apply mobile sensor networks to industrial inspection tasks that are too expensive or even impossible to be carried out using traditional industrial sensing technologies [3]. One such example is the PicoSmart project [4] that was started two years ago in the north of the Netherlands. Pipeline integrity management is a critical procedure to maintain the reliability and safety of industrial pipeline systems. Natural gas pipeline networks are usually probed by large cylindrical devices, called “pigs”, which are pushed through the pipes by the gas flow. Pigs collect sensing data about the working conditions of the pipes that they inspect and report the data to data processing servers at some outlets after they are retrieved. Pigs are quite large, and they can only move along reasonably clean pipes. It is usually difficult form them to pass sharp bends. For these reasons, pipeline networks with smaller diameters, with dirty inner surfaces or with irregular turns cannot be pigged, and are inspected by other less reliable and more costly ways or even not inspected at all.

The main idea of the PicoSmart project is to deploy a swarm of autonomous robots as a mobile sensor network. Each sensor equipped robot is small and agile, and thus can move freely within a pipeline network while taking sensing measurements. The additional computational and maneuvering capabilities of each robot make it also possible for the robot to maintain and repair a faulted pipeline from the inside. Normal pipeline-inspecting pigs are large, but in the PicoSmart project a heterogeneous and dynamic group of small “pico pigs” is to be deployed. Different types of small robots as a group will be able to perform the same functions as regular pigs and may even have additional functionalities because of their modularized architectures. The implementation of such a flexible swarm requires various capabilities of the robots. For example, the robots need to learn to adapt, both as individuals and as a group; they need to stay as a cohesive group, and thus formation control becomes necessary. These two aspects have in fact been the focal points of our research.

One of the main technical challenges is how to coordinate such a network of mobile sensors effectively and efficiently [5], especially considering the complex and changing hazardous environment inside the natural gas pipeline networks. Note in particular that each autonomous robot is usually constrained by its limited sensing and communication ranges and thus has only access to local information, while the coordination task within the sensor network is evaluated at an overall level [6]. This challenge is exactly the motivation for the research work to be presented in this paper. We aim to develop a framework for the design of novel distributed control and coordination strategies for networks of mobile sensors carrying out gas pipeline inspection tasks. Such theoretical developments are further verified through digital simulations and physical experiments. In other words, our work serves as primitive, but essential steps for establishing an integral approach to attack the distributed coordination problems for the application of mobile sensor networks for gas pipeline inspection tasks. Note that the results obtained in this paper are not confined to the application to natural gas pipeline inspection tasks. They are potentially useful for other pipeline networks for the delivery of oil, water, sewage, chemicals and so on. In the long run, they are also applicable to other physically enclosed harsh environments, such as water tanks, wings of air vehicles, mines and even outer space [7,8].

In this paper we utilize the combination of an integral approach and an object-oriented hierarchical structure. Although there are existing works combining ideas from robotic swarms, artificial intelligence and object-oriented design, the work presented in this paper distinguishes itself by a new research context of natural gas pipeline inspection tasks and a novel integration of the related ideas in the above fields in the same framework of mobile sensor networks.

The rest of the paper is organized as follows. The formulation of the problem and the proposed general solution framework is presented in Section 2. In Section 3, we present a simulated study on how to design algorithms for mobile sensors to learn to adapt to an unknown environment and benefit from collaborating with its peers. A robotic experimental study for coordinating mobile sensors to maneuver as a team with local information is then discussed in Section 4. We make concluding remarks at the end of the paper.

## 2. Problem Formulation and Proposed Integral Approach

### 2.1. Problem Formulation

We consider a team of sensor equipped autonomous robots [9] that operate in a given natural gas pipeline network. Each robot is powered by batteries and can move freely inside the pipeline network. It can detect within its sensing range a predefined fault on the wall of a tube and then record it in its memory. The information about the detected fault, together with other related sensing data, can be processed by the computational unit of the robot for further usage. The raw data and the processed information can be communicated between robots when they are within communication ranges of one another. Each robot has a distinguishable identity in the group, which is used to initiate or plan a coordinated action among a subset of the group members. The gas pipeline network environment is assumed to be stationary, *i.e.*, no significant changes of its condition happen while the robots are doing the inspection job. However, the robots have no prior knowledge about the working condition of the pipeline network. In other words, the robots have to rely on their sensing, computation and communication capabilities to build up their knowledge about the environment that they are working in.

The problem to be studied is how to control and coordinate the robots’ sensing, computation and communication activities so that the robots can cover the given pipeline network with the highest possible accuracy and the shortest possible execution time. Here, the definition for accuracy and execution time may vary for different networks and different robots at hand. Specific examples for such definitions are provided later in the case studies.

### 2.2. Proposed Approach

As has been discussed before, several factors make this problem especially difficult to handle. Firstly, each robot has to learn to adapt to the unknown pipeline network. Although each robot is mobile, it is still constrained by its limited power supply. So the usual random search approach is not a preferred choice, and more sophisticated learning strategies have to be adopted. Secondly, each robot has only limited sensing capabilities. As a result, there is no global information available to the group, and they have to share information with one another [10]. Thirdly, each robot has only limited communication capabilities. If a robot wants to remain as a member of the cohesive group, it has to coordinate with the other members and cannot make decisions or take actions completely independently [11]. In short, when dealing with the formulated problem, one is faced with a distributed, coordinated sensing task within a physically constrained unknown environment.

The approach proposed in this paper on how to deal with the problem is an integral one. We first build a theoretical framework that embeds the learning and adapting functionalities of the robots in an object-oriented hierarchical structure. Such a framework clearly defines the modularized functions in an industrial inspection task and leaves sufficient flexibility when being implemented in different mobile sensors in different natural gas pipeline networks. Then we consider the digital simulations and physical experiments as two empirical components complementing the theoretical analysis. Thus, the three components integrate together to comprise a solution to the formulated problem. The full development of the complete solution to the problem is beyond the scope of the paper, but we in this paper use two examples to show the effectiveness of the proposed solution. In what follows, we emphasize the object-oriented structure [12] that is key to the development of the theoretical framework.

### 2.3. Object-Oriented Design

The learning capabilities at the individual and the group levels, and the flexible and effective coordination among the mobile sensors, emerge in our design as a result of the object-oriented principle in the proposed solution framework. Here, object orientation means that the overall system’s functionality is split up into parts with the associated data structures and interactions. Correspondingly, each part is called an Object. An Object has Properties, Methods and Events. A Property of an Object describes an attribute of the Object or a data set that it retains. A Method is an action that an Object can perform, which can be called (initiated) by another Object. An Event is a self-induced action that triggers a change inside or outside an Object. An Object’s interface may include the Object’s Properties, Methods and Events and is typically also associated with a Service Level Agreement for that interface. The interface insulates the inside of an Object from its surroundings and can be used to introduce coupling planes, data abstraction and hardware abstraction layers. Objects are modularized and are usually organized hierarchically. They can encapsulate one another, inherit some others’ functions and even be polymorphistic.

Object-oriented design can be implemented in the individual mobile sensors [13]. An example is the Method “moving forward”, which is implemented internally within a robot as “rotating wheels to move a few centimeters ahead”. This allows the re-use of the programming codes and also allows the use of standard off-the-shelf components. Object orientation also introduces abstraction. An example is that a robot in an underwater environment can still have the same Method “moving forward”, but internally this is implemented by a different command of “waving the tail fin to swim forward”. Another advantage of using an object-oriented architecture is that it increases flexibility. For example, bacteria may appear in gas pipelines and to prevent them from corroding the tube walls’ coating, and they can be exterminated by a specialized robot installed with a radiating gamma ray source. Such a specialized robot can change its role from a searching robot to a bacterium-killing robot simply by using other internal Objects. A network of sensors with different sensing capabilities can share the same Objects, and the variation among sensors increases the robustness and flexibility at the group level.

The mobile sensor network as a whole also obeys the object-oriented design principle. The overall group also acts as an Object. For example, after the group has explored the given pipeline network for a sufficiently long period of time, a “time-out” event may trigger a finishing inspection method of the Object of the whole network. We want to point out here that such a network Object has a special form because there is no central command and no single point of contact. Hence, its methods can only be carried out in parallel using cooperative information propagation mechanisms.

In the following section, we use a case study to show how the mobile sensors’ learning and adapting abilities are achieved using the proposed framework, especially how objected-oriented design is implemented.

## 3. Simulated Learning Strategy

In this section, for a mobile sensor network operating in a natural gas pipeline network, we show a three-level architecture for the goal of achieving learning at the individual level, the sub-network level, and the network level.

### 3.1. Model

#### 3.1.1. Learning at the Individual Level

**Figure 2.**
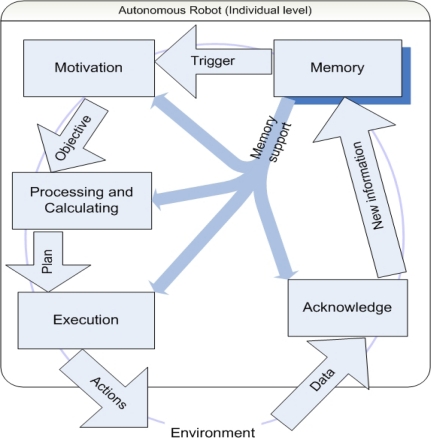
Information flow for an autonomous robot.

In Figure 2, we show a diagram indicating the signal flow within a modularized individual autonomous robot, for which we also develop a cognitive framework [14].

The learning capability of a single mobile sensor and of a network of such sensors is based upon the interactions among a number of Objects, which in turn interact with the environment. A central Object is Memory. New information about the environment is acquired via sensing and communication, then it is acknowledged and stored in Memory. A second Object, Motivation, drives the robot. Its function is defined in terms of striving for its goals by obtaining high rewards. The Motivation Object is triggered by changes originated in the Memory Object. It defines objectives and provides them to the Processing and Calculating Object, which uses rules and computations to come up with a plan for action. The Execution Object is able to convert this plan into real actions that the robot can take. These actions change the environment of the robot, which in turn triggers various sensors of the robot. This leads to new data to be processed by the Acknowledge Object. Thus a closed loop is formed.

**Figure 3.**
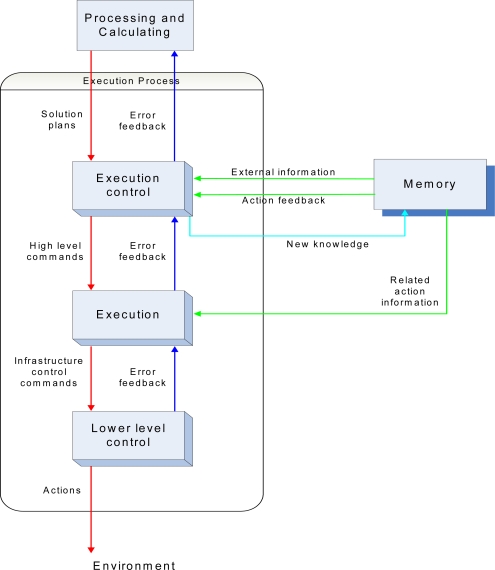
Execution Object within an autonomous robot.

The information flow thus forces the processing of the information, which leads to the execution of predefined actions realizing the robot’s goal of accurate and efficient sensing. We now discuss in more detail about the Execution Object in Figure 3 below.

This figure shows the Execution Object in Figure 2 in more detail. The Execution Object consists of a number of lower level Objects that interact with themselves and with external Objects. Its three internal Objects perform the desired execution in a hierarchical way and provide error feedback. Moreover, the three Objects receive additional required information from the Memory Object. The error feedback obtained while processing the received plans can lead to new knowledge, which is passed back to the Memory Object for storage.

#### 3.1.2. Simplifications and Assumptions

A number of simplifications and assumptions are made in the simulation. It is assumed that the amount of bacteria in gas pipelines has a direct relationship with the amount of weak spots in the walls of these tubes. The background colors of the tubes are used to simulate different bacterium levels and white spots represent weak spots. A robot is assumed to sense both the background color and the white spots, but to make the learning more challenging, we assume that taking measurements of white spots requires much more energy consumption than measuring the bacterium level.

In a darker-colored tube, more white spots appear on average and this average number is known to the robot. The simulated pipeline tube has four different background colors. The mobile sensors search for white spots over fixed intervals and probe the background color continuously.

#### 3.1.3. The Working Cycle of a Mobile Sensor

Each mobile sensor is designed according to the autonomous individual architecture shown earlier in Figure 2. When it moves in a straight line along the direction of the pipeline, it records the color of the tube and stores its speed. The robot checks its previous performances evaluated by accuracy and efficiency indices defined below every time it enters an area with a different color. The performance index *R_a_* for accuracy is defined by

(1) $$R_{a}=\{\begin{array}{cc} A & \mathrm{if}\;A\geq 0.7\\ A-C_{a} & \;\;\;\;\;\;\;\;\mathrm{if}\;A<0.7 \end{array}$$

where *A* is the proportion of the number of detected weak points over the total number of the weak points, *C_a_* is a chosen penalty constant when the accuracy is too low. The performance index for the efficiency is denoted by *R_t_*, which is inversely proportional to the time spent, namely the longer the execution time, the lower the *R_t_*. We further set a saturation bound for *R_t_*:

(2) $$R_{t}^{}=\text{min}(S,S_{\text{max}})$$

where *S_max_* is used as a penalty for excessive speeds. A robot’s Motivation Object aims for the highest possible performance and consequently achieving the highest reward. The total reward *R* for each robot *i* is then

(3) $$R^{i}=R_{a}^{i}+R_{t}^{i}$$

The robot updates its speed by looking into its memory about its past speed profiles in the areas with the same color. It tries to improve its performance following a deterministic rule modified by a randomization:

(4) $$S^{i}(t+1)=S^{i}(t^{*})*(1+\sigma )\begin{array}{cc} & t=0,1,2... \end{array}$$

(5) $$S^{i}(0)=S_{\text{min}}$$

where

(6) $$t^{*}=\underset{0\leq \tau \leq t}{\text{arg max}}\;R_{\tau }^{i}$$

and *σ* is uniformly distribution in [−*χ_i_*, *χ_i_*] with 0 < *χ_i_* < 1. Here, *χ_i_* can be taken as the “temperament” of the robot, and as a result of the introduced randomness, a “hot-tempered robot” tends to have large variations in its speed.

The process of learning is accelerated when a mobile sensor encounters a peer, and then compares the best performances of the two in the past in different areas. This information sharing procedure builds upon the individual learning layer and constitutes the second layer of learning, which is the learning within subgroups. We summarize these in the cycle of a mobile sensor described in the following figure.

The differences of the learning strategies are encoded in the levels of randomness in the speed update rules. This attempts to ensure that the performance of the group as a whole is not trapped at possible local minima. When a task run finishes, the mobile sensors gather at the service station to get recharged. Their performance data are collected and used for the design of better update rules, e.g., the probability distribution of each sensor’s randomness, and this can be taken as the learning at the group level.

### 3.2. Simulations

The simulations are carried out using the Webots software developed by Cyberbotics. The properties of each Object, such as shape, color, texture, mass, friction and so on, are configured completely through the Webots interface. Each mobile sensor is programmed as a two-wheel robot with a color-sensitive camera and its controller is programmed using the built-in integrated development environment together with the third party development environments using C++.

#### 3.2.1. Learning at the Individual Level

For a cold-tempered robot, its accuracy remains high due to its low speed and there is not much space for the improvement of its performance. An exception is the reward for the darkest color, since increasing speed implies missing spots.

**Figure 8.**
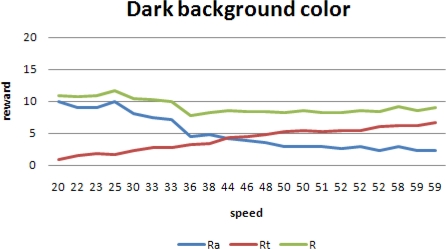
Conflicting *R_a_* and *R_t_* for dark background.

Figure 8 shows the runs with the darkest background color. A higher speed implies a higher *R_t_*, but since more spots are missed, *R_a_* is reduced.

The randomness in the speed update is set to be high in a hot-tempered robot. This gives rise to large variations in the robot’s speed, resulting in large variations in the robot’s performance:

Despite these large variations, a slow increase in the robot’s performance is observed, especially for the areas with lighter colors. Areas with a darker color have relatively more weak spots and correspondingly when moving fast through these areas, a mobile sensor may easily miss weak spots and the performance evaluated by *R_a_* worsens. This effect is less obvious for areas with lighter colors, which explains the obvious improvement in the performance in these areas.

#### 3.2.2. Learning at the Sub-Group Level

Here we present the simulation results for the case when two mobile sensors with different randomness meet each other.

A sudden rise in the average speeds is shown in this figure. Clearly the robot with lower randomness learns from the other robot and as a result its performance also improves significantly.

When focusing on one specific background color, the second level of learning becomes even more obvious when comparing the robots with different tempers.

Immediately after the learning at the sub-group level, the reward for the cold-tempered robot rises significantly. Also visible is the much higher spread in reward for the hot-tempered robot, which has already been shown earlier in the learning at the individual level.

Plotting reward against speed provides another view of the results.

When comparing the speed scales of both of the figures, it is clear that a cold-tempered robot does not pursue high speeds, but does get better rewards for higher speeds. Rewards for the darkest background are an exception for this as already observed before, since robots tend to miss spots at higher speeds.

A hot tempered robot does try high speeds. The medium dark color (pink in the graph) shows a maximum of approximately 65. For the lighter colors there are less spots and the chance that spots are missed increases, resulting in a relatively large “penalty” on accuracy. This results in very low rewards and a large spread in rewards.

When two mobile sensors with different randomness meet each other and learn from their interaction, the effect can be seen clearly.

The maximum for the medium dark background color can still clearly be observed, but the spread around this maximum is reduced significantly.

#### 3.2.3. Learning at the Network Level

The learning at the network level involves the comparison of the performance of mobile sensors with different tempers. In the previous sections, we have shown that cold-tempered robots have slow learning and their average reward is significantly improved after learning from a hot-tempered peer, although this hot-tempered peer does not have a very good individual learning itself. The improvement is induced by collaboration.

Learning at the network level is implemented by collaboration at a higher level and using derivatives from learning at the sub-group level. The effectiveness of the first level of learning is the speed of individual learning, which can be measured by the slope of the reward curve. So the effectiveness of individual learning is a derivative of the reward in time. Similarly, the effectiveness of the sub-group level of learning is exhibited through the amount of sudden improvement in reward as a result of the communication with the peer.

When varying the temperaments of the members of the group, variations in effectiveness are observed. We have analyzed these results and adjusted the tempers of the robots in our simulations.

Similarly, the timing for sub-group learning has an impact on its effectiveness. Sub-group learning involves learning from a peer by exchanging information, and this is more effective when both peers have more time to learn individually.

The derivatives from those first and second levels of learning are the temperaments of members of the group and the schedule to update sub-group learning. The analysis of the effectiveness of the first and second levels of learning is then utilized to modify the temperaments, and update the sub-group learning. This is in fact the third level of learning, which improves the effectiveness of the group as a whole.

Since the implementation of the third level of learning uses the derivative of the information from the first two levels, it requires more information as input. This implies that implementing learning at the network level requires more robots, a bigger testing field, longer running time, and more frequent information exchange and collaboration. Implementing this upscaling makes learning in the network level an effective activity of the collaborative group as a whole.

Through these simulations, we have shown that object-oriented learning at the three different levels can be implemented. In the next section, we continue our study using a robotic testbed. What we want to show is that a specific distributed coordination task, formation control [15], is indeed achievable in a lab environment where not all the external influences are predictable.

## 4. Experimental Formation Control

The PicoSmart project uses a heterogeneous and dynamic swarm of autonomous robots to perform certain tasks as a group. This requires the mobile sensors to move cohesively and it is therefore of special interest to design distributed control algorithms to have the mobile sensors to move in formations [16]. We consider the challenging case when the only available information available for an autonomous robot to maintain its relative position in a formation is the measured distances to its neighboring peers. Such range measurements are obtained by range sensors.

### 4.1. Model

We assume that:

1. there is no global coordinate system;
2. the robots have the ability to map their own motion within their own coordinate system;
3. the robots have the ability to repeatedly or continuously measure the distance to any neighboring robots.

Suppose there are altogether *n* sensors in the team. Let *p_i_* ∈ ℝ^2^, 1 ≤ *i* ≤ *n*, denote the position of sensor *i*. Let *d_ij_* denote the distance between sensors *i* and *j* and d^*^_ij_ denote the desired distance between them. In order to measure the closeness of a particular formation to a desired formation, we define a Lyapunov function [17] as follows:

(7) $$W(p_{1}(t),p_{2}(t),p_{3}(t),...,p_{n}(t))=\sum_{(i,j)\in \varepsilon }{\left(d_{\mathrm{ij}}^{2}(t)-d_{\mathrm{ij}}^{*2}\right)}^{2}$$

where ε is set of all the pair-wise distance constraints. The goal of formation control is to drive every sensor towards a target relative position in the team whose distance to the neighboring sensors are exactly the same as the desired values. In other words, the goal is achieved when the Lyapunov function is minimized.

To achieve this goal, each robot may localize its leaders’ positions in its own coordinate system using range-only measurements. To implement movements in a formation, a robot needs to follow its leader robots. Since we have assumed that a robot can continuously measure the distances between itself and its neighbors, it can thus sense their relative motions, according to which it can deduce who are its followers. We choose to use a distributed stop-and-go maneuvering strategy. By this we mean that a robot moves towards its target position, which satisfies all the distance constraints with respect to its leaders, and then stop when it gets to the target position. Under this scheme, a robot only moves when its leaders are stationary and its followers are at their target positions.

**Figure 16.**
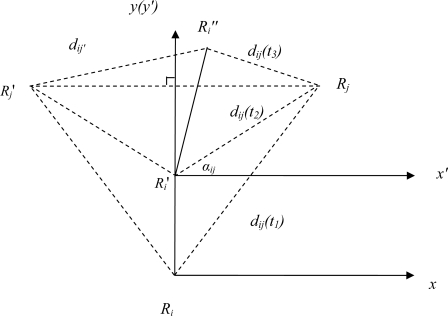
Localization.

As shown in Figure 16, a robot *i* is following another robot *j* and robot *i* is localizing its own position *R_i_* and robot *j*’s position *R_j_* in its local coordinate system. Towards this end, robot *i* takes two range measurements with respect to robot *j* when the latter is kept stationary. Using the distances *d_ij_(t_1_)* and *d_ij_(t_2_)*, and the distance between robot *i*’s two consecutive positions, robot *i* can localize the position of robot *j* within its coordinate system through planar geometric calculations. With the distance between *R_i_* and *R_i_*′, *d_ij_(t_1_)* and *d_ij_(t_2_)*, from the law of cosines, robot *i* first calculates the bearing *α_ij_* and the two candidate positions for robot *j*, which can be further uniquely determined when one more indicated range measurement is made. To be more specific, robot *i* moves to the third location *R_i_*″ and takes the third range measurement *d_ij_(t_3_)*. The position of robot *j* can be finally determined by comparing *d_ij_(t_3_)*, *d_ij′_* and *d_ij_*. Since each robot can map its own trajectory, after the above localization procedure, robot *i* knows its current position *R_i_*″ and position of robot *j*. Robot *i’s* next action is then to move to reduce the error of the distance between robot *i* and robot *j*.

As is apparent, this algorithm requires that during the motion period of a follower, the leader has to keep stationary. Thus, when applying this algorithm to more than two robots, a movement scheduling is needed to determine the sequence of motion of each robot. We set the event triggers in the stop-and-go maneuvering to be the following:

- Move activator: one robot starts its motion only when all its leaders are stationary, and the distances between itself and its followers are equal to the desired distances respectively;
- Stop activator: one robot finishes its motion only when all the distances between itself and its leaders are equal to the desired distances respectively.

Thus the stop-and-go maneuvering consists of the following three components:

1. the stop-and-go scheduling that determines the sequence of motions of each robot within the group;
2. the localization with range-only-measurements, using which each robot localizes the positions of its leaders and itself in its own coordinate system;
3. and the motion to reduce the value of the Lyapunov function.

The various components of the stop-and-go maneuvering strategy are all designed and implemented in an object-oriented way: the triggers are implemented as Events, communication and activation are implemented as Methods and distances are Properties of the robot Objects.

### 4.2. Experiments

A testbed has been developed to test the proposed formation control strategy. Three e-Puck robots are utilized, which are connected to PCs through Bluetooth communications. The goal of the experiment is to test the accuracy of the stop-and-go maneuvering strategy.

This strategy is implemented using the same object oriented architecture that is described in Figure 2. The difference is that this time the hierarchical structure of the sub-Objects within the Motivation Object and the Processing and Calculating Object of the robots focus on the stop-and-go maneuvering strategy instead of the three levels of learning.

**Figure 17.**
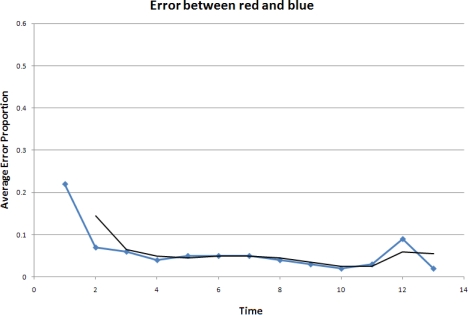
Error between the red and blue robots.

**Figure 18.**
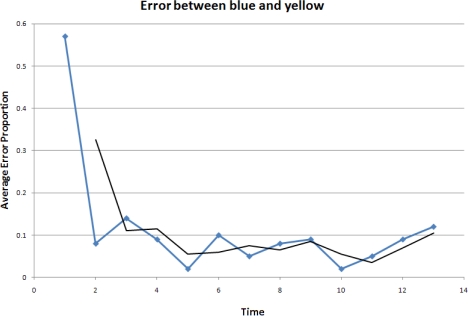
Error between the blue and yellow robots.

**Figure 19.**
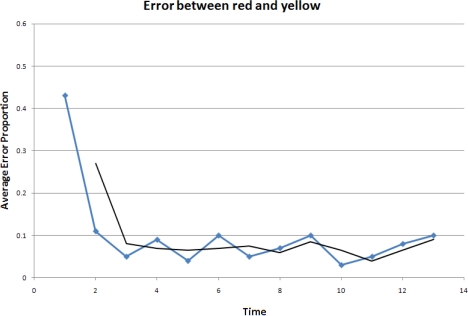
Error between the red and yellow robots.

In the experiment, three robots, labeled red, yellow and blue, start from random initial positions. The robots begin moving according to the model described in the previous section and a formation is formed over time. The accuracy is evaluated by the difference between the real formation and the desired formation. Here, we choose to use the errors between the pair-wise distances in the real formation and those in the desired formation. The results are shown in Figures 17, 18 and 19.

It is clear that overall the relative errors are between 10% and 5%. In our experiments, the red robot follows the blue one, and the yellow one follows both the red and the blue robots. As a result, the calculations done by the red robot are less complex and the yellow robot is much more sensitive to the error propagation and accumulation. This explains why the errors between the red and blue robots are lower and form a smoother curve than those when the yellow robot is involved.

The experimental results show that the errors in the pair-wise distances in the controlled formation are at an acceptable level even in this challenging scenario where only range measurements are available to the robots. The applied stop-and-go maneuvering strategy using the object-oriented framework has reasonably good performances.

## 5. Concludsions

An integral approach has been discussed in this paper in order to deal with the distributed coordination problem for mobile sensor networks in industrial inspection tasks. We have built a theoretical framework embedding the learning and adaptation capabilities in an object-oriented hierarchical structure. The framework has been further implemented and tested using digital simulations and physical experiments.

In the simulations, we have enabled the mobile sensors to learn at the individual, sub-network, and network levels through the object-oriented design of the working cycle of each mobile sensor. In the experiments, we have carried out the formation control task using range-only measurements. A distributed stop-and-go maneuvering strategy is obtained following again an object-oriented approach. The rules and strategies that each mobile sensor adopts in both the simulations and experiments are simple and intuitively straightforward; however, such design has proven to be effective through the simulations and experiments. This is an encouraging step towards applying mobile sensor networks in harsh industrial environments.

Next we plan to extend the results obtained in this paper to more general settings with less simplified assumptions. Utilizing the resources provided by the PicoSmart project, we will make a joint effort with our industrial partners to look into the research problems that arise in real gas pipeline networks. We identify sensing and communication errors, environment perturbations and uncertainties, and human operator evolvement as the main challenges in the future.

## Figures and Tables

**Figure 1.**
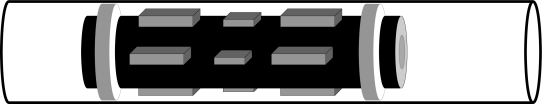
A “pig” inspecting a pipe.

**Figure 4.**
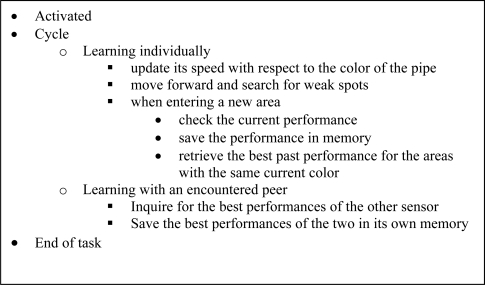
Working cycle of a mobile sensor.

**Figure 5.**
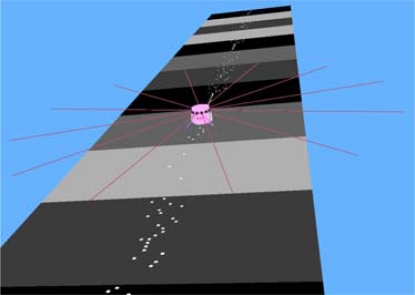
A snapshot of a moving mobile sensor.

**Figure 6.**
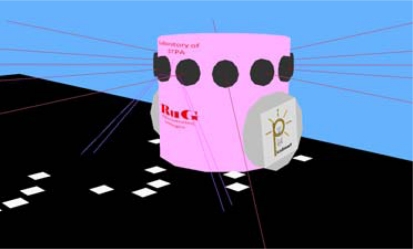
Details of a mobile sensor.

**Figure 7.**
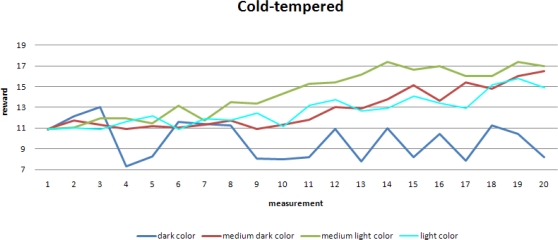
Performance of a robot with a lower randomness in its speed update.

**Figure 9.**
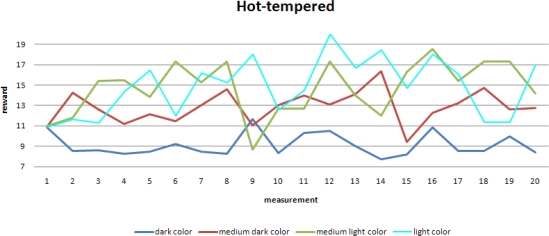
Performance of a robot with a higher randomness in its speed update.

**Figure 10.**
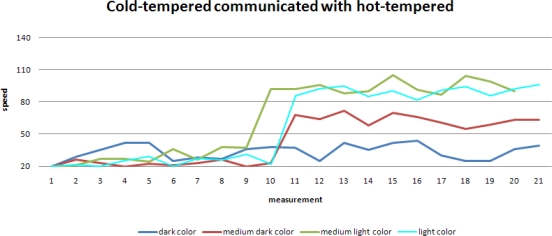
Speed of a mobile sensor with lower randomness learns from its peer with higher randomness.

**Figure 11.**
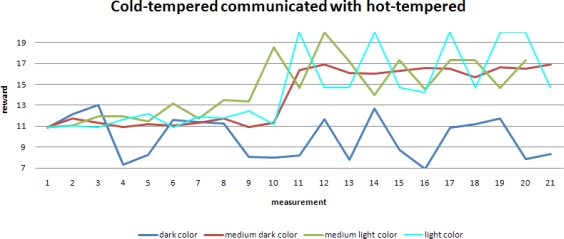
Performance of a mobile sensor with lower randomness when learning from its peer.

**Figure 12.**
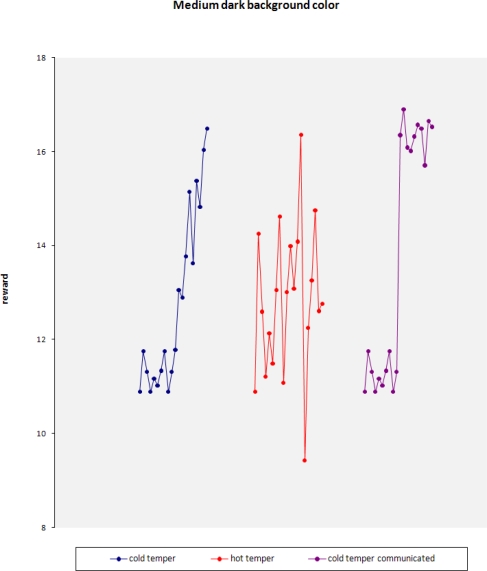
Observed reward for robots with different tempers (constant background color).

**Figure 13.**
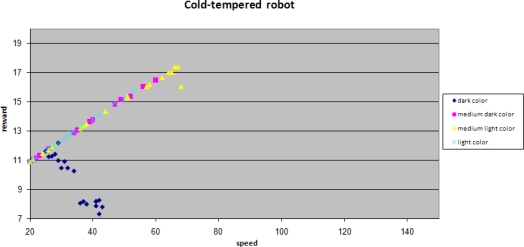
Performance of a cold-tempered robot against its speed.

**Figure 14.**
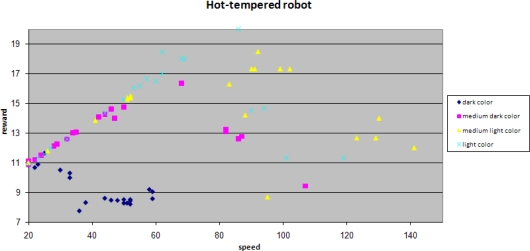
Performance of a hot-tempered robot against its speed.

**Figure 15.**
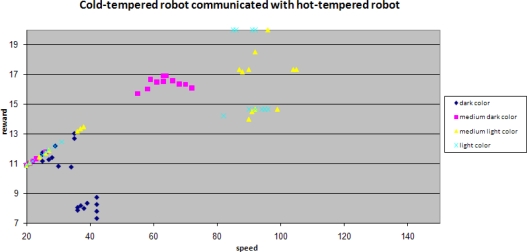
Performance of a cold-tempered robot against its speed when learning from its peer.
